# New Approach for the Detection of Sub-ppm Limonene: An Investigation through Chemoresistive Metal-Oxide Semiconductors

**DOI:** 10.3390/s23146291

**Published:** 2023-07-11

**Authors:** Arianna Rossi, Elena Spagnoli, Francesco Tralli, Marco Marzocchi, Vincenzo Guidi, Barbara Fabbri

**Affiliations:** 1 Department of Physics and Earth Sciences, University of Ferrara, Via Saragat 1/C, 44122 Ferrara, Italy; elena.spagnoli@unife.it (E.S.); francesco.tralli@unife.it (F.T.); vincenzo.guidi@unife.it (V.G.); 2 Sacmi Imola S.C., Olfactory Systems, Via Selice Prov.le, 17/a, 40026 Imola, Italy; marco.marzocchi@sacmi.it

**Keywords:** chemoresistive gas sensors, limonene, metal oxides, sensor arrays, selectivity, cosmetics, food industry

## Abstract

R-(+)-limonene, one of the major constituents of citrus oils, is a monoterpene that is widely used as a fragrance additive in cosmetics, foods, and industrial solvents. Nowadays, its detection mainly relies on bulky and expensive analytical methods and only a few research works proved its revelation through affordable and portable sensors, such as electrochemical and quartz crystal microbalance sensors. In response to the demand for effective miniaturized sensing devices to be integrated into Internet of Things systems, this study represents a pioneering investigation of chemoresistive gas sensor capabilities addressed to R-(+)-limonene detection. An array of seven metal-oxide sensors was exploited to perform a complete electrical characterization of the target analyte. The experimental evidence allowed us to identify the WO_3_-based sensor as the most promising candidate for R-(+)-limonene detection. The material was highly sensitive already at sub-ppm concentrations (response of 2.5 at 100 ppb), consistent with applicative parameters, and it resulted in selective vs. different gases at a lower operating temperature (200 °C) than the other sensors tested. Furthermore, it exhibited a humidity-independent behavior under real-life conditions (relative humidity > 20%). Finally, the WO_3_ sensor also demonstrated a remarkable cross-selectivity, thus enabling its exploitation in cutting-edge applications.

## 1. Introduction

**Figure 1:**
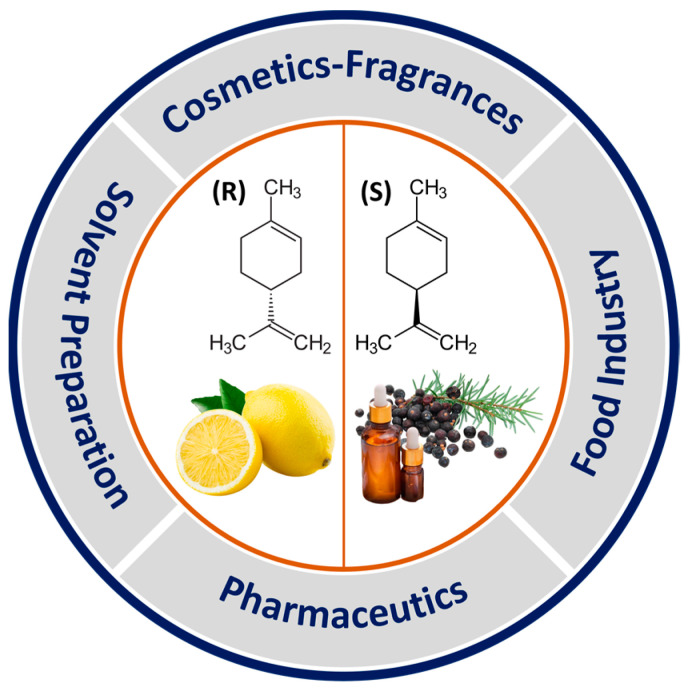
R-(+)-limonene and S-(+)-limonene optical isomers, respectively, and their applications.

Limonene, the ordinary and commercial name to indicate 4-isopropenyl-1-methyl-1-cyclohexene, is an organic chemical compound belonging to the family of terpenes. As a chiral molecule without any plane of symmetry, it exists in nature mainly in the form of two enantiomers: R-(+)-limonene and S-(+)-limonene [1,2,3,4,5]. The former is certainly the most common and is present in large quantities in the essential oils produced in *Citrus* fruit peels, such as lemons (hence the name), oranges, and limes, but also in cumin, dill, and bergamot oils. On the other hand, S-(+)-limonene is less frequent, can be found in the oily resins of some conifers and gives off a strong turpentine odor (Figure 1) [1].

The availability of the pristine material, essentially created through distillation or centrifugation from plant waste, together with its appreciated organoleptic properties, allows it to find space in numerous industrial applications. For instance, thanks to its pleasant citrus smell and characteristic taste, limonene is often exploited in the food and beverage industry as a flavoring and preservative element (e.g., in fruit juices, baked goods, sweets, chewing gums, ice creams, etc.). In addition, it is widely used in beauty and personal care products, such as soaps, shampoos, perfumes, and cosmetics in general. Finally, it can also be found in household products (detergents, cleaning products, room fresheners), in certain types of natural solvents, and as the active principle in ecological pesticides [1,3,5]. R-(+)-limonene is a volatile organic compound (VOC) that is also frequently found in indoor air. As a pure substance, it is considered to be a chemical with fairly low toxicity and safe for human beings if inhaled in small doses, such as at typical indoor concentrations [2]. Many studies also report promising positive effects: limonene is rapidly metabolized, it has no mutagenic risks, and it is therapeutic against viruses, diabetes, inflammation, and other diseases [1,3,5].

However, it has also been largely demonstrated that it interacts with ozone and some free radicals present in the air, oxidizing and producing volatile substances that contribute to air pollution [6,7]. These include secondary organic aerosols and photochemical smog, which are reactive and show potent toxic effects, causing airway and skin irritation [1,8]. The time-weighted average threshold limit value (TLV-TWA) of R-(+)-limonene is about 30 ppm. Therefore, having at disposal a good monitoring system is important in order to keep R-(+)-limonene levels within potentially dangerous limits [9].

Lab-based analyses are the primary methods used to monitor limonene emissions. The laboratory techniques conventionally used to investigate and quantify limonene and other VOCs, in general, can be of various types: cyclic (CV) or differential pulse voltammetry (DPV), high-performance liquid chromatography (HPLC), gas chromatography coupled with mass spectrometry (GC–MS), and electrochemical impedance spectroscopy (EIS) [10]. The physical principles driving the operation of these methodologies are distinct from each other, and in terms of performance, GC–MS is among the most accurate and reliable tools for gaseous compound identification, establishing itself as the de facto standard for this field. However, all these solutions have several practical drawbacks that undermine their use on a large scale. For example, GC–MS requires heavy, bulky, and expensive equipment, specialized personnel, and complicated pre-treatment steps, thus making it unsuitable for real-time detections [4,10,11,12]. Therefore, it is clear that techniques of this type are far from practical for simple and rapid routine detections. Rather, methods featuring reduced costs for the equipment, reduced processing time, fast responses, and a reduced amount of solvents and samples are needed.

Nowadays, gas sensors represent the most affordable alternative to lab-based analyses for rapid and promising R-(+)-limonene detection. For instance, electronic nose (e-nose) systems have been explored in odor assessments due to their versatility and similarity to the human olfactory system [13,14]. However, e-noses require complex analysis and pattern recognition modules, resulting in a long training procedure [12]. The majority of studies on limonene sensors can be classified into electrochemical and quartz crystal microbalance sensors. Concerning electrochemical sensors [10,15], besides a short lifetime (one to three years) due to greater exposure to the target gas, their output signal (current) is proportional to the area of the electrode. Thus, avoiding a bulky size of the device means limiting the active surface exposed to the analyte. Moreover, they suffer from interference, and it is difficult to identify failure modes unless very advanced methods of monitoring are used. Recently, sensors based on a quartz crystal microbalance (QCM) coated with organic conducting polymers have been studied and employed in e-noses [16]. QCM sensors, widely used in food detection, are affected by temperature and humidity, and they have a high detection limit. They also suffer from the mass change of the sensor surface, since the thickness of their layer limits its capabilities to adsorb more water molecules, and consequently of the device to measure higher dew points [11,16]. Accordingly, QCM sensors are typically designed to operate in low moisture applications. This represents an impediment for real-life applications that are not able to leave humidity out of consideration. Moreover, QCM sensors feature a strongly limited lifetime [11], which is too short for potential commercialization. Finally, despite a high reactivity, the conductive polymers used as functional materials in QCM are expensive and their poor solubility limits large-scale sensor production.

Although the gas sensing performance of solid-state devices based on inorganic materials are usually lowered in humid conditions, this category of sensors still allows a fair compromise to measure moistures in a wide range of concentrations with an adequate accuracy in the lower end. Chemoresistive gas sensors based on metal-oxide (MOX) semiconductors are commonly known for their sensitivity, low cost and power consumption, scalable production, durability, and integrability in Internet of Things (IoT) systems. MOX materials for gas sensing applications were an important focus in recent years since they can be tailored to achieve desired surface-to-volume ratios and morphologies, so as to attain various levels of performance. However, the number of studies explicitly focusing on MOXs for limonene detection is extremely limited. They are mostly integrated into more complex and expensive olfactory systems, such as e-noses, and they usually need to be supported by refined laboratory methodologies with the sole purpose of discriminating between fingerprints of different VOCs [4,17]. Despite their popularity and widespread use in a myriad of sectors, an exhaustive and systematic study of their sensing capabilities towards R-(+)-limonene is hitherto lacking [15]. Therefore, in order to probe their potentiality in R-(+)-limonene detection, in this work we decided to perform a complete sensing performance characterization of a chemoresistive sensor array based on seven diverse MOX materials.

## 2. Materials and Methods

*Sensing materials.* Considering the plethora of MOXs investigated in the literature so far, the aim of this study was to explore some of them as sensing materials specifically for R-(+)-limonene detection. We started from well-known, pure MOXs, i.e., SnO_2_, WO_3_, and ZnO, then we moved to doped ones, i.e., Pd:SnO_2_ and Au:SnO_2_, and finally we assessed more elaborate solid solutions, such as (Sn,Ti,Nb)_x_O_2_ and WO_40_Sn_60_O_x_. The selection of these seven nanostructured MOXs was based on the experience and understanding of our sensor laboratory [18,19,20,21,22,23].

*Sensor production.* The selected materials were firstly synthesized in powder form via simple, reproducible, and previously optimized chemical processes. Then, they were mixed with α-terpineol, ethyl cellulose, and silica to form homogeneous pastes. Concerning the organic solvent sources, the liquid component (α-terpineol) was from Sigma-Aldrich, with a mixture of isomers of ≥96%, whereas the solid component (ethyl cellulose) was from Sigma-Aldrich with a viscosity of 5% *w*/*w* solution in 80:20 toluene:ethanol by weight at 25 °C. The organic precursors can be included in a total amount ranging from 50 to 80% of the mass depending on the consistency of the compound to be obtained for an optimal deposition, while silica constitutes a fraction between 0.5 and 1%. Its role is to strengthen the adhesion both among the nanostructures of the functional materials themselves and between the sensing layer and the alumina substrate [24]. The resulting pastes were then treated by sonication and screen-printed onto alumina substrates (substrate area 2.54 × 2.54 mm^2^ and thickness 250 μm, Metallux) [25]. These were equipped with interdigitated gold electrodes on the front side (Figure S1a, Supporting Information), which provide electrical contacts for measuring the conductance of the sensing material, and with a platinum heater on the back side (Figure S1b, Supporting Information) to thermally activate the sensing layer at the desired working temperature. The screen-printing mask mesh was 250, and the thickness of the films and the active area were 20–30 μm and 1 mm^2^, respectively. Finally, the semiconducting MOX films were sintered in air for 2 h at 650 °C in order to (i) increase the grain interconnectivity required for electronic conductance, (ii) improve the stability of the structure in thermal activation, (iii) remove any residues of organic precursors, and (iv) enable the functionality of silica improving the adhesion both among the nanostructures of the functional material and between the sensing layer and the substrate.

At last, the electrodes and the heater were connected to the electronic system via the pins of a commercially available TO39 support. The connection between the elements on the substrate and the pins of the support was made using 0.06 mm diameter gold wires, welded using thermo-compression through a wedge wire bonder.

*Experimental setup.* The sensors were electrically characterized in a sealed test chamber in which temperature and relative humidity (RH%) were controlled by a commercial Honeywell HIH-4000 (Figure S2, Supporting Information). The devices were exposed to controlled concentrations of gaseous mixtures. Synthetic air (20% O_2_ and 80% N_2_) and target gases were fluxed from certified cylinders (N 5.0 degree of purity, Sapio) through mass-flow controllers (Brooks), achieving a total flow rate of 500 sccm. The filling time of the test chamber was estimated to be about 1 min and 15 s, as it depends on its size and geometry and on the velocity of the gas flow. The test chamber was placed into a climatic system to maintain a constant temperature of 25 °C and ~2 RH%. Humid air was obtained by injecting a fraction of the total synthetic air flux into a gas bubbler filled with deionized water [26]. The final RH% values were cross verified using a commercial humidity sensor. Power suppliers (Aim TTi) and a multimeter (K2000 (Keithley)) were used to provide the necessary electric current to the sensor heater and to read the electrical conductance of the sensing film, respectively. A constant bias of 5 V was applied to the two interdigitated gold electrodes. The architecture of the acquisition circuit is based on an operational amplifier (OA). Since the voltage values *V_in_* and *V_out_* are connected at the ends of the sensor resistor *R_s_* and applied load resistor *R_f_*, respectively, then the gain is given by *V_out_*/*V_in_* = −*R_f_*/*R_s_*. Accordingly, the expression for the sensor conductance *G_s_* is givens is as follows:

(1) $${Sensor\;Conductance\;(G}_{s})=\frac{1}{R_{s}}=-\frac{V_{out}}{R_{f}\cdot \;V_{in}}$$

Considering the n-type of the semiconducting sensing films, their response was calculated as in Equations (2) and (3), where *G_air_* is the sensor conductance in dry air and *G_gas_* is the conductance under selected gas exposure [23].

(2) $$Response\left(R\right)=\frac{{(G}_{gas\;}-G_{air})}{G_{air}}for\;reducing\;gases$$

(3) $$Response\left(R\right)=\frac{{(G}_{air}-G_{gas})}{G_{gas\;}}for\;oxidizing\;gases\;$$

*Sensing performance.* The sensor performances were proven by making a complete electrical characterization. First, the best operating temperature of each MOX film was investigated considering their response to 1 ppm of R-(+)-limonene at increased working temperatures, ranging from 200 to 400 °C. Then, in order to study the sensitivity, the films were exposed to several concentrations of R-(+)-limonene (100, 200, 300, 500, 1000, and 5000 ppb) under dry conditions. To verify the influence of possible interfering compounds, they were also exposed to two concentrations (5 and 10 ppm) of different volatile analytes, such as acetone, acetaldehyde, and ethanol. Gases and concentrations were chosen in order to cover chemical species that might be relevant for cosmetics and similar above-mentioned applications. For instance, the effective detection and monitoring of targeted gas in cosmetics, namely ethanol and R-(+)-limonene, are essential for product quality and human skin care. The selectivity coefficient (*k_s_*) [27] of the sensors for R-(+)-limonene detection was calculated as in Equation (4), i.e., by the ratio of the response values to R-(+)-limonene and the interfering gas at 5 ppm, respectively.

(4) $${Selectivity\;Coefficient\;(k}_{s\;})=\frac{R_{R\text{-}(+)\text{-}limonene\;}}{R_{interfering\;gas}}$$

By comparing the responses to several individual gas injections, it is possible to evaluate the ability of the sensors to detect different analytes and, in turn, indirectly estimate their selectivity for the target gas. However, these tests do not allow assessment of the influence of gaseous competition mechanisms for the active sites on the surface of the materials when more than one compound is present simultaneously in the environment. Then, the cross-selectivity of the sensors vs. 5 ppm of R-(+)-limonene starting from consecutive exposures to 10 ppm of acetone, acetaldehyde, and ethanol was estimated.

Humidity is a very common interferent because water vapor can dissociate when interacting with the film, affecting its conductance and generating –OH groups that limit the adsorption site on the surface [19]. To explore its effect on the sensor performance, 500 ppb of R-(+)-limonene was injected into the gas chamber at different percentages of relative humidity, from 2 up to 74 RH%.

Finally, the repeatability of the 7 sensors at their best working temperature was also tested under 1 ppm of R-(+)-limonene in dry conditions. The response (*τ_res_*) and recovery (*τ_rec_*) times were calculated as the times needed to attain 90% of the steady-state response and required to switch back to 90% of the baseline value, respectively [28].

*Investigation on surface reactivity.* The array of sensors examined in this work aimed to explore different promising classes of intrinsic and extrinsic active sites for R-(+)-limonene detection. To date, it is hard to experimentally observe and measure the overall number of each surface atom family. Consequently, it is challenging to explain why some MOXs have higher sensitivity than others toward this target molecule.

Some hints on the surface composition can be deduced from the sensing properties towards a series of sample analyte gases. For example, the sensitivity to NO_2_ was found to be enhanced with electron donor sites, e.g., oxygen vacancies [29]. On the other hand, the sensitivity to CO is dependent on the surface reducibility of the MOX surface, which reflects the concentration of oxidizing sites [29]. Then, NO_2_ and CO were chosen as probe molecules for the investigation of the film surface characteristics at the sensors’ optimal temperature for R-(+)-limonene sensing. Gas concentrations of 25 ppm and 3 ppm for CO and NO_2_, respectively, were selected according to their relative TLV-TWA [30,31].

## 3. Results

**Figure 2:**
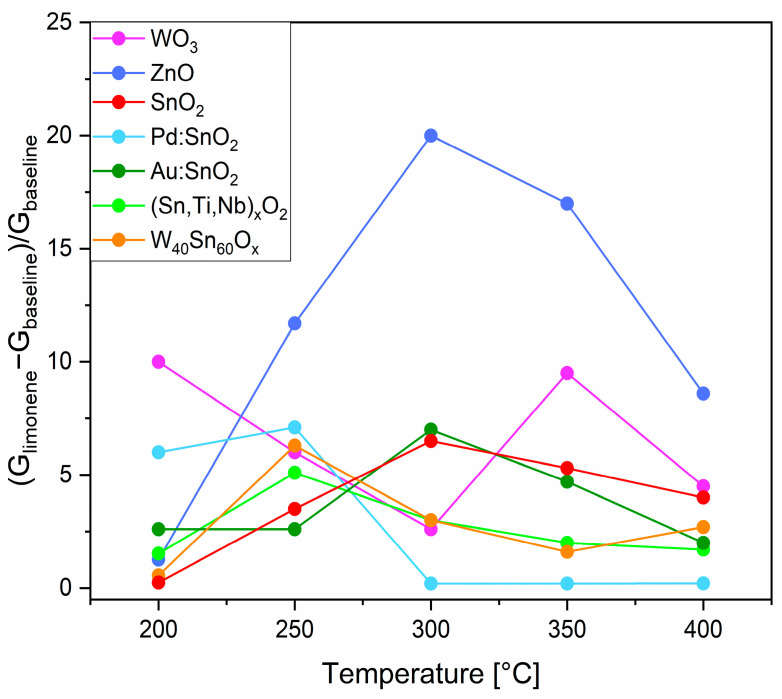
Response vs. working temperature of the sensors to 1 ppm of R-(+)-limonene.

The **optimal working temperature** of the sensors was determined by measuring the conductance variation before and after injection of 1 ppm of R-(+)-limonene in dry conditions at different working temperatures within 200–400 °C (Figure 2). The response of the sensors based on ZnO, SnO_2_, Pd:SnO_2_, Au:SnO_2_, (Sn,Ti,Nb)_x_O_2_, and W_40_Sn_60_O_x_ initially improved with increasing temperature, reached a maximum, and then reduced with further increases in temperature. This behavior is generally observed in chemoresistive MOX sensors [32]; indeed, each material exhibits its highest response level at the specific working temperature that maximizes its catalytic activity vs. R-(+)-limonene, promoting surface redox reactions. Above the optimal value, the rate of gas desorption became appreciable, lowering the sensor response [20,33,34]. Differently from the other sensors, WO_3_ displayed two maxima responses at 200 °C and 350 °C, suggesting two distinct operative conditions for optimized detection of the analyte, probably characterized by different active sites on the surface for the chemisorption of R-(+)-limonene. According to the literature [35], the active species resulting from the ionosorption of oxygens commonly are O_2_^−^ in the range 100–200 °C and O^−^/O^2−^ at higher temperatures [35]. In the temperature range between the two optimal values (200 °C and 350 °C), an unfavorable competition between different reaction mechanisms of the analyte absorption and desorption would lead to a decrease in WO_3_ sensing capabilities. The optimal temperature for each sensor is reported in Table 1 and was chosen hereinafter for its electrical characterization. For WO_3_, the working temperature of 200 °C is preferred to meet the requirement for low power consumption in portable and IoT applications.

Figure S3 (Supporting Information) displays the raw data signal of the sensors operated at their best working temperature to show their detection kinetics. The increase in *V_out_*, correlated with the conductance of the n-type semiconducting film according to Equation (1), implies that R-(+)-limonene is acting as a reducing gas, releasing electrons into the conduction band.

**Figure 3:**
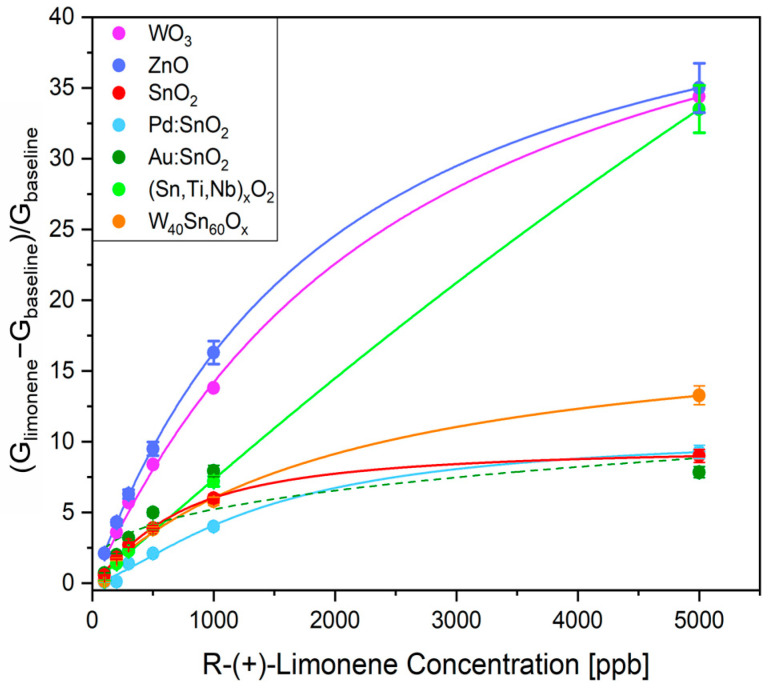
Responses of the films vs. 100, 200, 300, 500, 1000, and 5000 ppb concentrations of R-(+)-limonene. The calibration curves of WO_3_, ZnO, and (Sn,Ti,Nb)_x_O_2_ fitted with Langmuir isotherms are shown as thick lines. The dash lines are designed only to track the trends of SnO_2_, Pd:SnO_2_, Au:SnO_2_, W_40_Sn_60_O_x_.

The **sensitivity** was investigated by exposing the sensors to 100, 200, 300, 500, 1000, and 5000 ppb of R-(+)-limonene. Figure 3 highlights that WO_3_ and ZnO sensors at each tested concentration exhibited higher responses than the others and they did not saturate at the highest value (5000 ppb). Therefore, they can detect R-(+)-limonene gas in a wide range of concentrations that are typically found in many of the applications mentioned. Moreover, despite a low response below 1000 ppb, the (Sn,Ti,Nb)_x_O_2_ sensor demonstrated good sensitivity at high concentrations. The calibration curves of WO_3_, ZnO, and (Sn,Ti,Nb)_x_O_2_ were fitted with Langmuir isotherms [36]. The parameters *a* (maximum adsorption capacity), *b* (ratio of the adsorption and desorption rates), and *c* (equilibrium concentration) together with the coefficient of determination *R*^2^, are listed in Table S1 (Supporting Information). In particular, the goodness of the fit was found to be inappropriate (*R*^2^ lower than 0.9) for the Au:SnO_2_ sensor. Due to the limited number of measurements, the adoption of a different functional form for the regression (e.g., allometric fit) would result in strong overfitting. Therefore, no other function could be used for the construction of a calibration curve that could be correctly employed in device programming.

**Figure 4:**
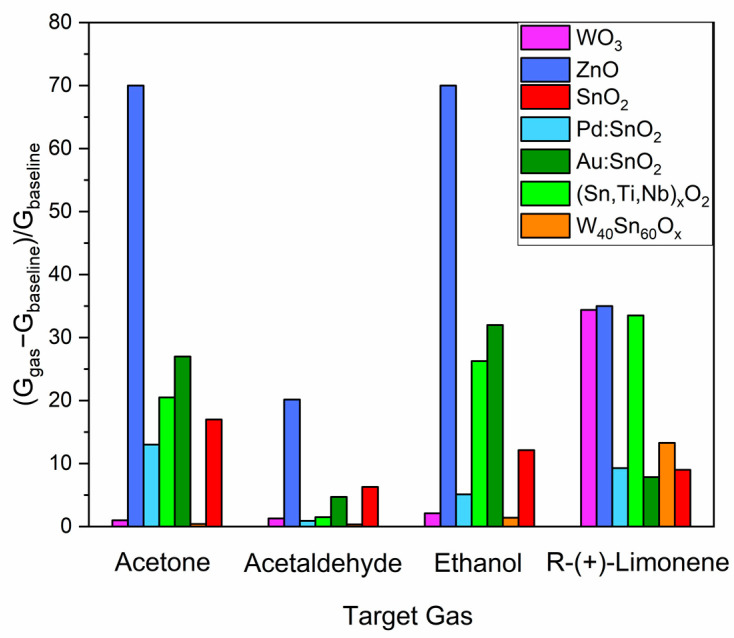
Response of the sensors vs. 5 ppm of acetone, acetaldehyde, ethanol, and R-(+)-limonene in dry air.

The **selectivity** of the sensors is another important parameter due to the heterogeneous gaseous composition occurring in real applications such as food processing, solvent production, and especially in cosmetic and fragrance preparation. In particular, this work sought to distinguish the selectivity toward various functional groups, including terpenes, alcohols, aldehydes, and ketones, which are classes of gases frequently present in the aforementioned applications. Figure 4 shows the sensors response vs. 5 ppm of R-(+)-limonene, acetone, acetaldehyde, and ethanol. All samples exhibited an n-type sensing behavior, namely, a conductance increase during exposure to reducing gases, see Figures S4–S7 (Supporting Information). The selectivity coefficients *k_s_* estimated for R-(+)-limonene detection are reported in Table S2 (Supporting Information). Despite ZnO’s higher reactivity to R-(+)-limonene than all other sensing materials, this sensor was not selective since the responses to ethanol and acetone were even higher than those of the target gas. The selectivity of the most commonly used SnO_2_ and of the other materials based on its modifications (Pd:SnO_2_, An:SnO_2_, and (Sn,Ti,Nb)_x_O_2_, W_40_Sn_60_O_x_) were likewise inadequate. On the other hand, WO_3_ demonstrated an effective ability to discriminate against R-(+)-limonene among other interfering gases.

**Figure 5:**
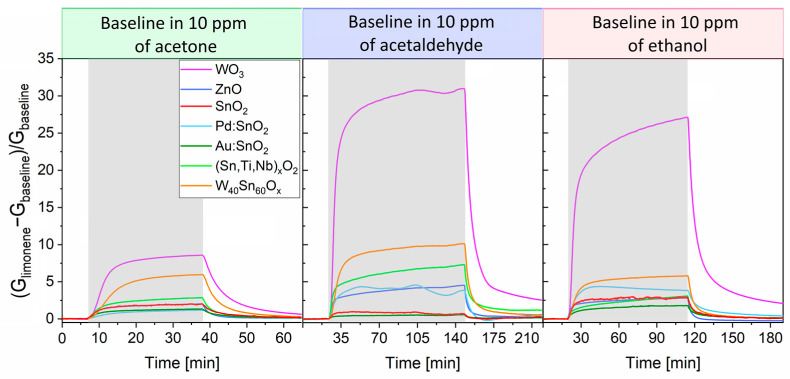
Sensor responses towards 5 ppm of R-(+)-limonene in an atmosphere of 10 ppm of acetone, acetaldehyde, and ethanol. Gray intervals represent the period of limonene injection. The duration of the measure depended on the time required for the majority of the sensors to achieve the steady state in presence of the target gas.

The **cross-selectivity** of the sensors vs. 5 ppm of R-(+)-limonene in the presence of 10 ppm (baseline) of acetone, acetaldehyde, and ethanol was analyzed and displayed in Figure 5. The response of those based on ZnO, SnO_2_, Pd:SnO_2_, Au:SnO_2_, (Sn,Ti,Nb)_x_O_2_, and WO_40_Sn_60_O_x_ significantly fell in presence of the interferents. Remarkably, considering the behavior to 5 ppm of R-(+)-limonene in dry conditions (~35), WO_3_ seemed unaffected by ethanol and acetaldehyde as the response values were almost maintained. Only in the presence of acetone did the WO_3_ sensor response (~8.5) decreased by approximately four times compared to other measurements.

**Figure 6:**
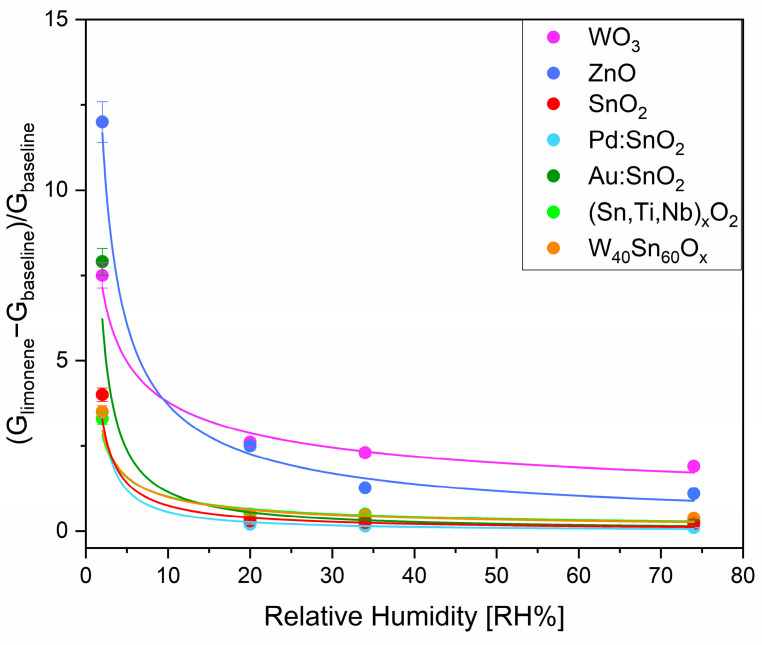
Humidity effect on sensor responses towards 500 ppb of R-(+)-limonene. The error bars correspond to 5% of the instrumental error.

**Figure 7:**
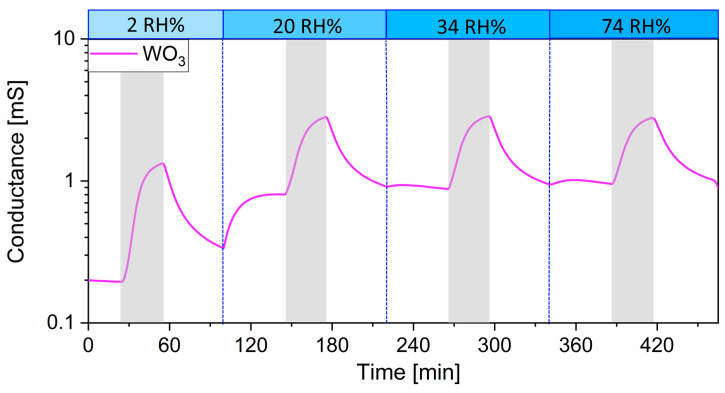
Dynamic response of WO_3_ sensor to 500 ppb R-(+)-limonene under different humidity conditions. Gray intervals represent the period of gas injection. The light blue bars show the value of RH%.

The **influence of humidity** on the sensing performance vs. R-(+)-limonene was examined by comparing the responses of the seven sensors against the target analyte (500 ppb) while they were exposed to increasing concentrations of water vapor (2–74 RH%). As shown in Figure 6, the responses significantly plummet up to 20 RH%, decreasing by 65% for WO_3_ and 80% for ZnO, while inhibiting the detection of SnO_2_, Pd:SnO_2_, Au:SnO_2_, (Sn,Ti,Nb)_x_O_2_, and W_40_Sn_60_O_x_. At humidity levels higher than 20 RH%, the response of ZnO to R-(+)-limonene only slightly diminished, while it remained constant for the WO_3_ sensor. Due to the promising properties of the latter, previously reported in this work, and since it is the least influenced by humidity, we further focused on WO_3_ conductance behavior (Figure 7). Firstly, one can observe an increase in the baseline conductance of the sensitive film when the sensor was exposed to 20 RH% due to the homolytic dissociation of water, which reacted with the metal site and a lattice oxygen to form a terminal and a rooted hydroxyl group. The latter acted as a surface donor, freeing an electron to the conduction band [27,37]. Secondly, this reaction mechanism also affected the catalytic properties of the film vs. R-(+)-limonene detection due to a change in the chemical composition of the surface by substitution of the oxygen ion active sites, present in dry conditions, with the newly formed hydroxyl groups. As a consequence, under wet conditions the response to the analyte decreased. Thirdly, the conductance was only slightly affected by humidity increases above 20 RH%, indicating that the surface was almost uninfluenced by additional rises in water vapor content. For this reason, in the same range of RH%, the baseline conductance and the response to R-(+)-limonene only marginally decreased, then were almost humidity independent. These characteristics can improve sensor accuracy and simplify calibrations [38].

The repeatability of the dynamic response vs. 1 ppm of R-(+)-limonene during three consecutive cycles was investigated and it is shown in Figure S8 (Supporting Information). The films exhibited a clear reversible interaction and good repeatability for detection over time. The response and recovery times are reported in Table 2. It was observed that they were similar for all sensors except for ZnO, which showed faster kinetics. Response and recovery times within minutes were already obtained for SnO_2_ and other materials in our previous works [19,23]. These parameters depended on the size and geometry of the chamber and on the gas flow velocity.

## 4. Discussion

**Figure 8:**
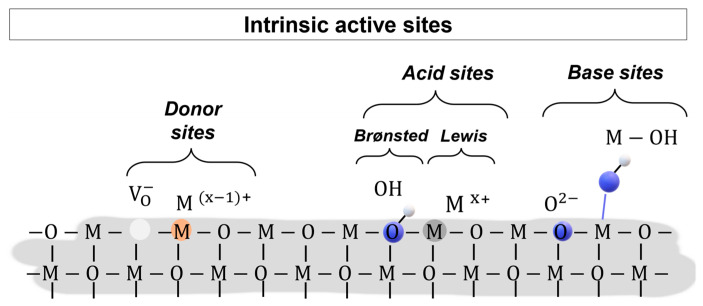
Two-dimensional schematic representation of a MOX surface with typical intrinsic active sites. From left to right: charged oxygen vacancies (V_O_^−^), partially reduced metal cations (M^(x−1)+^), hydroxyl Brønsted sites (OH), coordinately unsaturated metal cations (M^x−+^), oxygen anion (O^2−^), base hydroxyl site (M–OH).

Nanomaterials based on MOXs have been meticulously researched for use in chemoresistive gas sensors because their electronic properties are sensitive to chemical processes occurring at the surface. Indeed, redox reactions of oxidizing or reducing gas molecules control the thickness of the space-charge region of the grains composing the sensing film, therefore influencing its overall conduction. The selectivity vs. target gas, i.e., R-(+)-limonene, is ruled by the number and nature of the active sites at the MOX surface. Figure 8 provides a schematic two-dimensional representation of the intrinsic active sites at a pristine MOX surface. This includes coordinately unsaturated metal cations and oxygen anions, point defects (cationic and anionic vacancies, atomic interstitials, and dislocations), and –OH groups in hydrated MOX [29].

**Figure 9:**
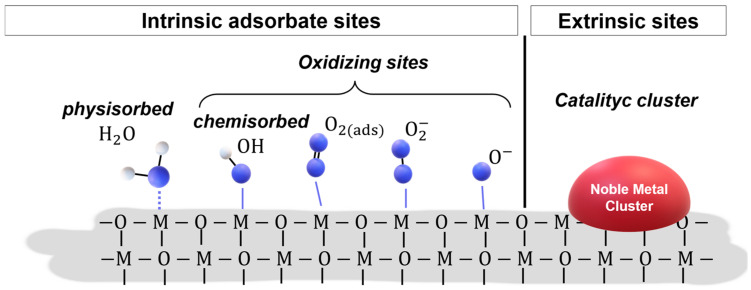
Two-dimensional schematic representation of a MOX surface with typical intrinsic adsorbate sites and extrinsic sites. Active sites on the surface, such as physisorbed water molecules, chemisorbed –OH groups, chemisorbed oxygen species (O_2_(ads), O_2_^−^, and O^−^), and noble metal clusters, are depicted in the Figure.

Moreover, spontaneous adsorption of oxygen and water vapor can generate intrinsic adsorbate sites, depending on the operating temperature (Figure 9) [29]. In fact, between 100 and 500 °C, the interaction of the sensing layer with atmospheric oxygen leads to ionosorption of molecular (O^2−^) and/or atomic (O^−^, O_2_^−^) species. In the present study, developed at an operating temperature ranging from 200 to 300 °C, oxygen anions such as O^−^ and O_2_^−^ dominate the ionosorption process. In addition to pristine structural and spontaneous active sites, new extrinsic active sites can be created via the introduction of additives: atoms, atomic groups, clusters, nanoparticles of metals, or metal oxides [29].

Each aforementioned surface site possesses a definite chemical behavior in terms of acidity/basicity, electron donor/acceptor attitude, and specific chemisorption capacity for probe molecules [29]. Such properties are described in the literature [29,39,40] and are derived from the nature of the MOX (cationic charge and size, metal–oxygen bond energy), its synthesis conditions, microstructure, additives, and operative conditions (e.g., sensor operating temperature). Therefore, to demonstrate the key role of the active sites in determining the selective interaction between sensing materials and gases of different nature, the sensor responses to probe molecules, i.e., 3 ppm of NO_2_ and 25 ppm of CO, are shown in Figures S9 and S10 (Supporting Information), respectively. The response of WO_3_ towards NO_2_ revealed a high reactivity. Contrarily, all other sensing materials were poorly reactive to this oxidizing gas. On the other hand, the WO_3_ response to CO was lower than that of ZnO and comparable to that of SnO_2_-based sensors. These results experimentally demonstrated a remarkable diversity in surface reactivity between WO_3_ and other materials, which was probably crucial for the better sensitivity of this sensor vs. R-(+)-limonene and, above all, its highly effective cross-selectivity. Indeed, from the response of WO_3_ vs. NO_2_, it can be deduced that its sensing performances were influenced by a combination of (i) donor sites (e.g., oxygen vacancies), (ii) Lewis acid states (unsaturated W^6+^), and (iii) preadsorbed oxygen species, which reacted with NO_2_ [29,41]. Instead, poor responses towards NO_2_ and competitive responses to CO occurred for ZnO- and SnO_2_-based sensors, indicating that their selectivity was mostly related to oxidizing sites [29].

Finally, under humid conditions, one has to consider that –OH groups produced by the dissociation of water molecules on the surface can act as interferents [27]. In particular, the interaction between –OH groups that behave as Lewis acid sites and surface oxygen anions that behave as Lewis bases promoted the adsorption of R-(+)-limonene in the WO_3_ film, and the sensor signal remained poorly affected by the presence of moisture (Figure 7).

In summary, the research conducted so far has highlighted different active surfaces of the materials towards redox reactions for R-(+)-limonene detection, which are more favorable in the case of WO_3_. Although to the best of our knowledge the reaction of R-(+)-limonene with chemoresistive sensor films is still unexplored, it is reported in the literature that the surfaces of MOXs catalyze the oxidation of the target molecule to epoxides, carvone, or carveol [42,43,44] which may further react with the film, contributing to the sensor response. The overall sensing mechanism should be rather complex, but in the end, it results in a reduction in the sensing layer and in an increase in its conductance.

## 5. Conclusions

The experimental evidence from sensitivity and selectivity tests, together with investigations on active sites using probe molecules, could lead future works on the development of modified MOX-based sensor arrays for the detection of R-(+)-limonene in a wide range of concentrations and in complex environments. ZnO was sensitive to several gases and, hence, it is not suitable for R-(+)-limonene sensing in gas mixtures. On the other hand, doping/loading and solid solution formation strategies have not proven to be effective in enhancing the performance of the most commonly used SnO_2_, which have low sensitivity and selectivity. Among the various nanostructured MOX films tested, only the one based on WO_3_ turned out to be suitable for the development of chemoresistive R-(+)-limonene sensors operating at low temperatures, demonstrating good sensitivity, cross-selectivity, and humidity-independent behavior for concentrations higher than 20 RH%. The investigation of the active sites using probe molecules highlighted the very different reactivity of WO_3_ compared to other MOXs, which was probably influenced by the presence of donor sites (e.g., oxygen vacancies) and Lewis acid states (unsaturated W^6+^). Such active sites are fundamental for R-(+)-limonene chemisorption on the surface of the material. Furthermore, since WO_3_ poorly detected polar interferents, such as acetone, acetaldehyde, and ethanol, it can be inferred that their surface adsorption sites were selective for apolar molecules, such as R-(+)-limonene. In future works, we will investigate the size effect of WO_3_ nanoparticles and the influence of the morphology on the sensing performance [18]. Moreover, the gas sensing mechanism occurring at the surface of WO_3_ will be studied using DC resistance measurements combined with diffuse reflectance infrared Fourier transformed spectroscopy (DRIFT) [45]. This advanced characterization technique could be also effective to comprehend the R-(+)-limonene cross-selectivity of the WO_3_ sensor with respect to a mixture including polar interferents, e.g., VOCs, addressing the typical applications in which it is necessary to detect this analyte.

## Figures and Tables

**Table 1:** Optimal operating temperature of the sensors vs. 1 ppm of R-(+)-limonene derived from Figure 2 and Figure S3 (Supporting Information).

Sensor	Optimal Working Temperature [°C]
WO_3_	200
ZnO	300
SnO_2_	300
Pd:SnO_2_	250
Au:SnO_2_	300
(Sn,Ti,Nb)_x_O_2_	250
W_40_Sn_60_O_x_	250

**Table 2:** Responses at the best working temperature of the sensors to 1 ppm of R-(+)-limonene and the response/recovery times deduced from the repeatability graph in Figure S8 (Supporting Information).

Sensor	Response ^1^	*τ_res_* [min]	*τ_rec_* [min]
WO_3_	10	18	50
ZnO	17	4	15
SnO_2_	6.5	17	46
Pd:SnO_2_	7.1	3	40
Au:SnO_2_	7	17	46
(Sn,Ti,Nb)_x_O_2_	5.1	24	57
W_40_Sn_60_O_x_	6.3	21	57

^1^ The responses were obtained using Equation (2).

## Data Availability

Not applicable.

## Supplementary Materials

The following supporting information can be downloaded at: https://www.mdpi.com/article/10.3390/s23146291/s1.
