# Supplementary material for: New Approach for the Detection of Sub-ppm Limonene: An Investigation through Chemoresistive Metal-Oxide Semiconductors
Source: Sensors (Basel). 2023 Jul 11;23(14):6291. doi: 10.3390/s23146291 (PMC10383529; doi:10.3390/s23146291)
Supplement: Supplementary file 1 [file sensors-23-06291-s001.zip › sensors-2454718-supplementary.pdf]

New Approach for the Detection of Sub-ppm Limonene: An Investigation through Chemoresistive Metal-Oxide Semiconductors

Arianna Rossi [1,*], Elena Spagnoli [1], Francesco Tralli [1], Marco Marzocchi [2], Vincenzo Guidi [1] and Barbara Fabbri [1,*]

[1] Department of Physics and Earth Sciences, University of Ferrara, Via Saragat 1/C, 44122 Ferrara, Italy; elena.spagnoli@unife.it (E.S.); francesco.tralli@unife.it (F.T.); vincenzo.guidi@unife.it (V.G.)
[2] Sacmi Imola S.C., Olfactory Systems, Via Selice Prov.le, 17/a, 40026 Imola, Italy; marco.marzocchi@sacmi.it
* Correspondence: arianna.rossi@unife.it (A.R.); barbara.fabbri@unife.it (B.F.); Tel.: +39-0532-974283 (A.R.); +39-0532-974213 (B.F.)

Materials & Methods

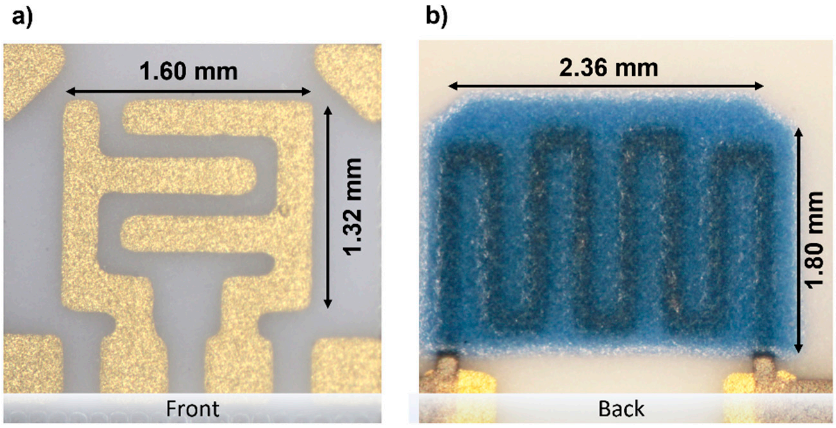

Figure S1. Images of a) the interdigitated electrodes on the front side and b) the platinum heater on the back side of an alumina substrate used for MOX sensors working in thermal-activation mode.

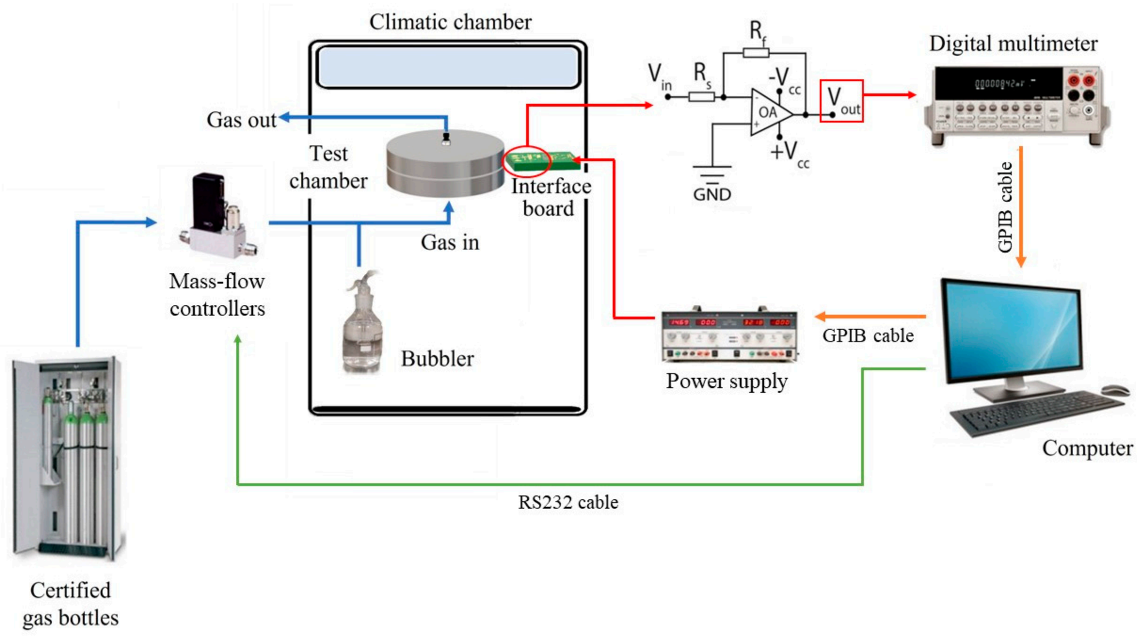

Figure S2. Schematic representation of the gas mixing system composed by certified gas bottles, mass-flow controller (MFC), bubbler for humidity control and sealed gas measurements chamber. Data acquisition system composed by suitable electronics, digital multimeter and power supply. Blue arrows are gas tubes, while red, orange, and green arrows are different types of electrical cables.

Experimental measurements

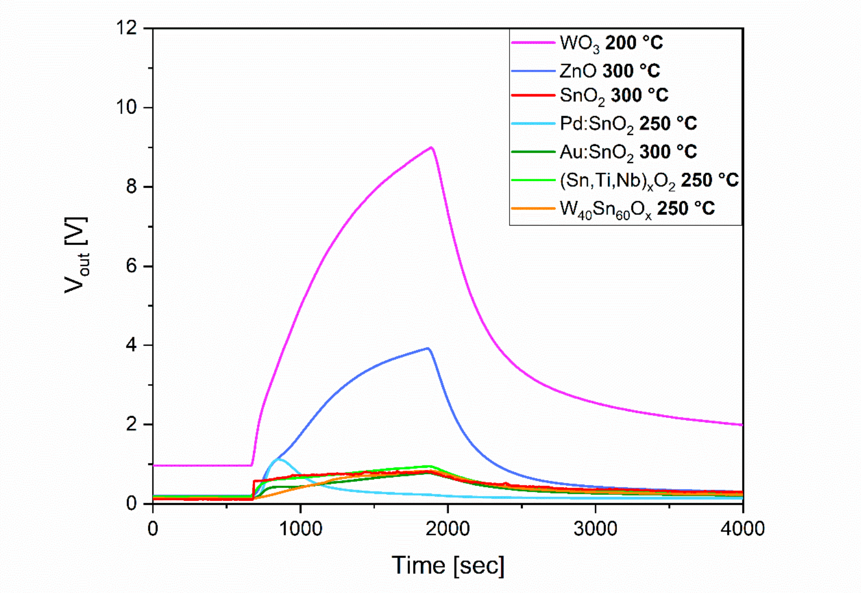

Figure S3. Dynamic raw signal to 1 ppm of R-(+)-limonene of the sensors at each proper best working temperature (**Table 1**).

Table S1. The parameters *a* (maximum adsorption capacity), *b* (ratio of the adsorption and desorption rates), and *c* (equilibrium concentration) of the calibration equations for WO_3, ZnO, $(Sn,Ti,NO)_xO_2$, SnO_2/Pd, WS60, and SnO_2 sensors. Uncertainties and estimated values are not rounded, but left as provided by the spreadsheet.

	Equation Langmuir $y = (a \cdot b \cdot x^{1-c})/(1+b \cdot x^{1-c})$			
Sensor	*a*	*b*	*c*	R^{2*}
WO_3	51.0 ± 10.6	(2.86 ± 2.41) × 10^{-4}	−0.0426 ± 0.168	0.999
ZnO	47.9 ± 0.800	(4.09 ± 3.53) × 10^{-5}	−0.0329 ± 0.0166	0.999
$(Sn,Ti,Nb)_xO_2$	192 ± 250	(3.02 ± 1.83) × 10^{-5}	−0.0387 ± 0.119	0.999
Pd:SnO_2	10.7 ± 1.18	(2.72 ± 4.59) × 10^{-5}	−0.452 ± 0.278	0.988
$W_{40}Sn_{60}O_x$	18.9 ± 4.35	(4.79 ± 4.68) × 10^{-4}	0.00423 ± 0.195	0.988
SnO_2	9.69 ± 0.289	(2.65 ± 1.21) × 10^{-4}	−0.264 ± 0.0800	0.997

* The coefficient of determination R^2 expresses the goodness of the fit.

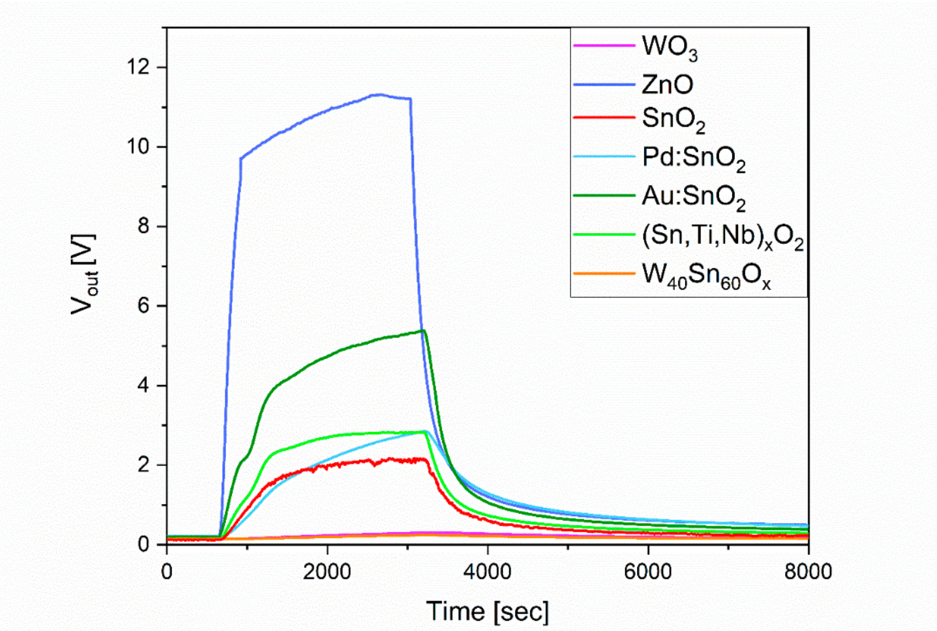

Figure S4. Dynamic raw signal to 5 ppm of acetone of the sensors at each proper best working temperature (Table 1).

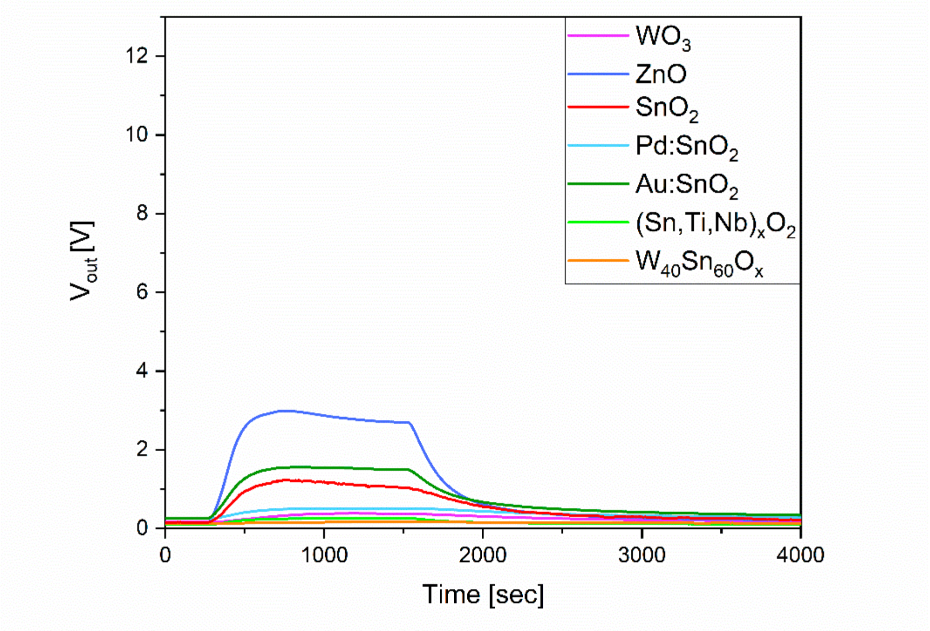

Figure S5. Dynamic raw signal to 5 ppm of acetaldehyde of the sensors at each proper best working temperature (Table 1).

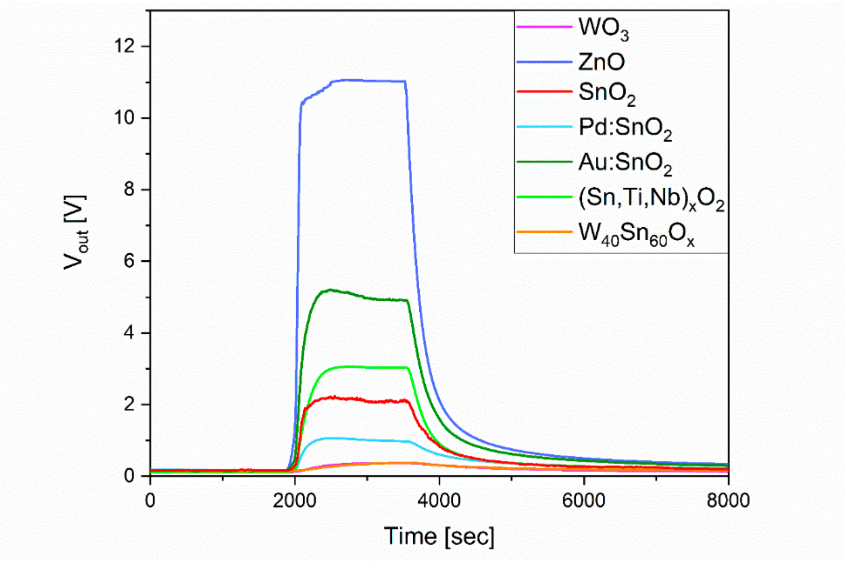

Figure S6. Dynamic raw signal to 5 ppm of ethanol of the sensors at each proper best working temperature (Table 1).

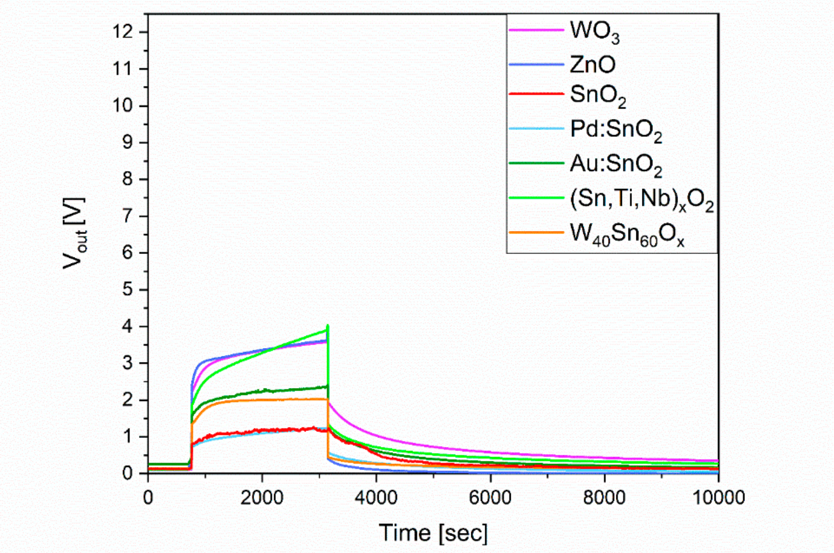

Figure S7. Dynamic raw signal to 5 ppm of R-(+)-limonene of the sensors at each proper best working temperature (Table 1).

Table S2. Selectivity coefficient (k_s) for 5 ppm of R-(+)-limonene calculated from responses reported in Figure 5 using Equation 3.

Sensor	k_{s1} (Acetone 5 ppm)	k_{s2} (Acetaldehyde 5 ppm)	k_{s3} (Ethanol 5 ppm)
WO_3	34.4	26.4	16.3
ZnO	0.500	1.73	0.500
SnO_2	0.530	1.43	0.740
Pd:SnO_2	0.710	10.3	1.81
Au:SnO_2	0.290	1.67	0.240
$(Sn,Ti,Nb)_xO_2$	1.63	22.3	1.27
$W_{40}Sn_{60}O_x$	33.2	40.2	9.48

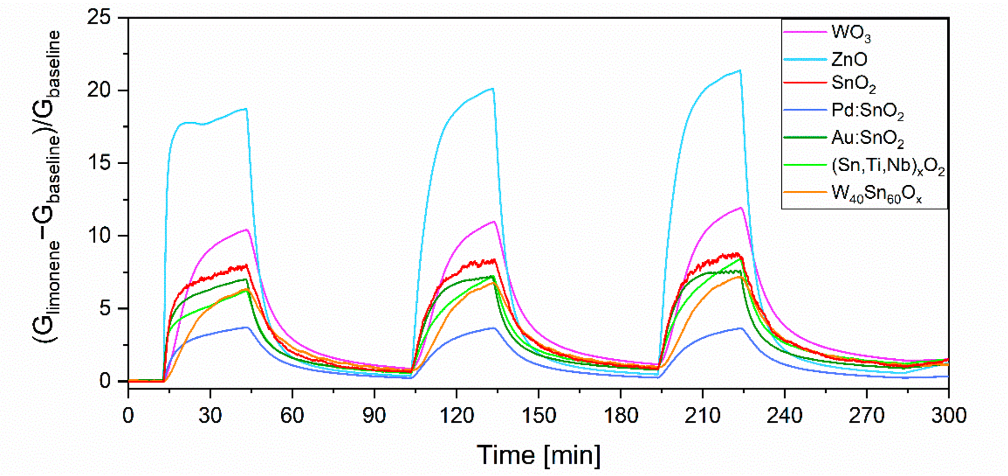

Figure S8. Responses to three-cycle injection of 1 ppm of R-(+)-limonene as a function of time.

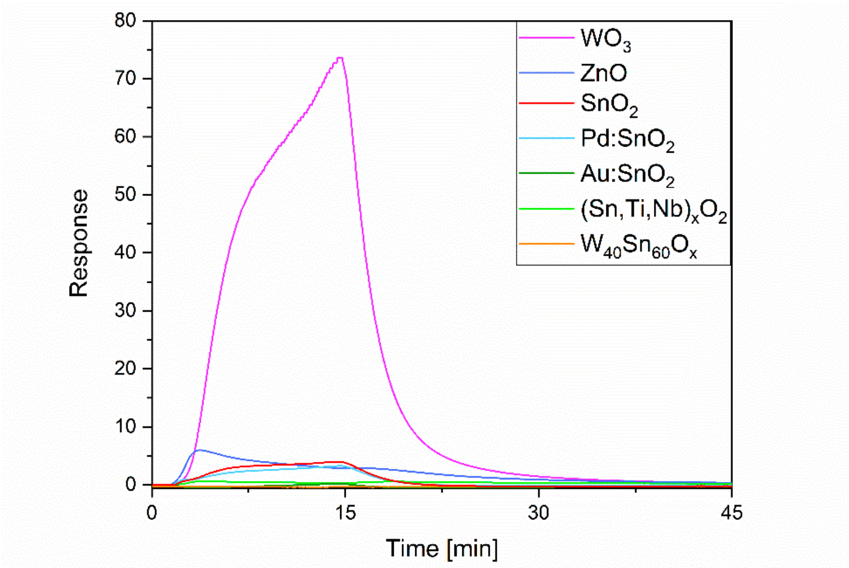

Figure S9. Response of sensors exposed to 3 ppm of NO_2, highlighting a high reactivity of WO_3 vs. this oxidizing gas.

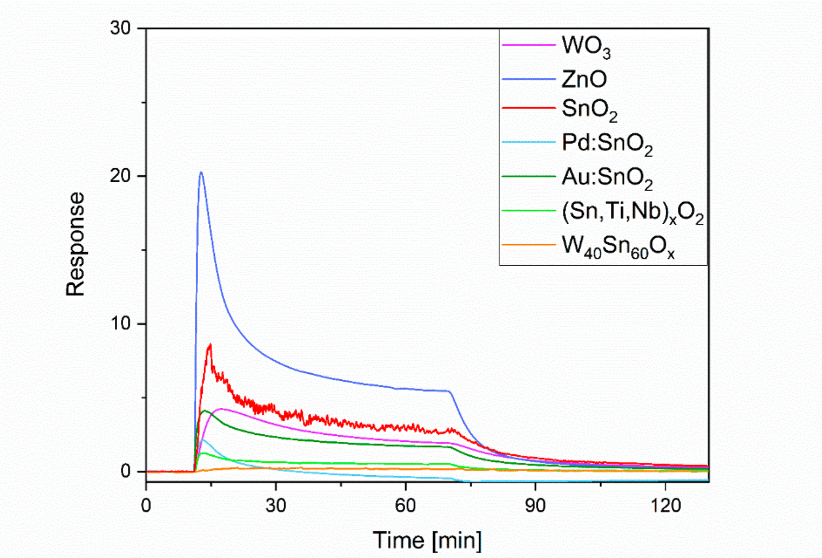

Figure S10. Response to 25 ppm of CO, displaying a general low reactivity of all the sensors. The reactivity of MOX towards this gas is commonly lower than that vs. other reducing gases and rises with increasing operating temperatures, which promote oxidizing sites formation. The optimal working temperature for R-(+)-limonene detection was probably too low to activate both pure and doped MOX.
